# Appetite Loss in Patients with Advanced Cancer Treated at an Acute Palliative Care Unit

**DOI:** 10.3390/curroncol31100452

**Published:** 2024-10-10

**Authors:** Elisabeth Hagen Helgesen, Ragnhild Ulevåg, Tora Skeidsvoll Solheim, Morten Thronæs, Gunnhild Jakobsen, Erik Torbjørn Løhre, Trude Rakel Balstad, Ola Magne Vagnildhaug

**Affiliations:** 1 Department of Clinical and Molecular Medicine, Faculty of Medicine and Health Sciences, Norwegian University of Science and Technology, 7030 Trondheim, Norway; tora.s.solheim@ntnu.no (T.S.S.); morten.thrones@ntnu.no (M.T.); erik.t.lohre@ntnu.no (E.T.L.); trude.r.balstad@ntnu.no (T.R.B.);; 2 Cancer Clinic, St. Olavs Hospital–Trondheim University Hospital, 7030 Trondheim, Norway; gunnhild.jakobsen@ntnu.no; 3 Centre for Crisis Psychology, Faculty of Psychology, University of Bergen, 5007 Bergen, Norway; 4 Department of Public Health and Nursing, Faculty of Medicine and Health Sciences, NTNU–Norwegian University of Science and Technology, 7030 Trondheim, Norway; 5 Department of Clinical Medicine, Clinical Nutrition Research Group, UiT The Arctic University of Norway, 9019 Tromsø, Norway

**Keywords:** appetite loss, cancer, palliative cancer care, symptom burden

## Abstract

Appetite loss is prevalent in patients with advanced cancer and negatively affects their quality of life. However, understanding of the factors associated with appetite loss is limited. The current study aims to explore characteristics and therapeutic interventions used for patients with and without appetite loss admitted to an acute palliative care unit. Patient characteristics and patient-reported outcome measures (PROMs), using the 11-point numeric rating scale (NRS 0–10), were registered. Descriptive statistics, independent samples T-tests and chi-square tests were utilized for data analysis. Of the 167 patients included in the analysis, 62% (104) had moderate to severe appetite loss at admission, whereof 63% (66) improved their appetite during their hospital stay. At admission, there was a significant association between appetite loss and having gastrointestinal cancer, living alone, poor performance status and withdrawn anticancer treatment. Patients with appetite loss also experienced more nausea, depression, fatigue, dyspnea and anxiety. In patients with improved appetite during hospitalization, mean decrease in NRS was 3.4 (standard error (SE) 0.27). Additionally, patients living alone were more likely to improve their appetite. Appetite improvement frequently coincided with alleviation of fatigue. Understanding these associations may help in developing better interventions for managing appetite loss in patients with advanced cancer.

## 1. Introduction

The terms appetite loss and anorexia describe a lack of desire to eat. Loss of appetite is frequent in people with severe and chronic illnesses such as cancer, chronic gastrointestinal, and kidney diseases [1]. Appetite loss is not measurable by biomarkers and is often best assessed by personal perceptions [2]. Consequently, there is a risk that loss of appetite may be underestimated and undertreated if assessed only by clinical evaluations [3,4].

Along with fatigue and pain, loss of appetite is one of the three most prevalent symptoms in patients with advanced cancer [5]. The consequences of reduced appetite include limited food intake, malnutrition, weight loss, impaired psychosocial wellbeing, and reduced quality of life (QoL) [6,7,8]. Loss of appetite is a negative predictor for survival [6,9,10,11]. In addition, reduced appetite and weight loss may affect the efficacy of anticancer treatments [12]. 

Elements contributing to loss of appetite are changes in taste and smell, and reduced motility in both the upper and lower gastrointestinal tract causing nausea, early satiety and constipation [12]. Additionally, other symptoms such as pain or reduced emotional wellbeing can cause appetite loss [13]. Some of these elements can be side effects of anticancer treatment and other pharmacological interventions. However, these elements may also be a direct consequence of tumor growth, or components of cancer cachexia. Cachexia is defined as a syndrome with loss of weight, muscle, and occasionally adipose tissue, which conventional nutritional support cannot reverse completely [14]. Cachexia is caused by both the tumor itself and the host’s response to the tumor. As a result, pro-inflammatory cytokines, peptides and hormones are released and affect receptors and neurons in the hypothalamus, causing a disturbance in energy homeostasis, increased inflammation, catabolism and often reduced appetite [12,15,16].

Despite its prevalence and negative impact, appetite loss is an understudied area where little is known about the association with patient-, tumor- and treatment-related factors. 

The objectives of the present study were to explore the characteristics of patients with advanced cancer and appetite loss compared to those without loss of appetite when admitted to an acute palliative care unit (APCU), and to investigate associations between therapeutic interventions and improvement of appetite. The results may ease the identification of patients at risk for appetite loss and potentially contribute to beneficial interventions. 

The research questions were:

(1) What were the differences in demographic and disease characteristics between palliative cancer patients with appetite loss and palliative cancer patients without significant appetite loss at admission to an APCU?
(2) What were the demographic and disease characteristics of patients who improved their appetite and those who did not during hospitalization?
(3) Which pharmacological interventions and changes in symptom intensities were associated with changes in appetite during hospitalization?

## 2. Materials and Methods

### 2.1. Study Design and Patient Population

The present article is a secondary analysis of a prospective longitudinal study investigating the provision of palliative care [17]. The study included adult patients with incurable cancer admitted to the APCU at the Cancer Clinic at St. Olavs Hospital, Trondheim University Hospital, Norway, between 15 January 2019, and 15 January 2020. All consecutive patients admitted to APCU during the study period were screened. 

In the current analysis, which explores appetite, all patients with a minimum of two self-reported registrations for appetite, one at admission and at least one follow-up, were identified and included. Each patient was included only once, and readmissions were therefore excluded.

The APCU consists of both an outpatient clinic and an inpatient ward with 12 beds and approximately 450 admissions every year. Patients are allocated to either an integrated care pathway or a palliative care pathway. In the integrated care pathway, the patients are followed by the APCU and the Department of Oncology in parallel while still receiving oncologic treatment. In the palliative care pathway, the patients no longer receive oncologic treatment. Patients with hematological, gynecological and lung cancer receive oncological and palliative treatment in their respective hospital departments and are only referred to the APCU when neuraxial pain management is needed. Further details about the observational study conducted at the APCU can be found in the main publication from 2021 [17].

### 2.2. Assessments and Data Collection

Details on data collection have been previously described [17]. Information was collected on demographics, cancer diagnosis, Eastern Cooperative Oncology Group (ECOG) performance status, care pathway and medications and other interventions performed during the hospital stay. Additionally, the patients reported subjective symptom intensity (SI) at admission and during hospitalization. Patient-reported outcome measures (PROMs) are an endorsed tool for collecting data about SI in patients in palliative care [18], and the Edmonton Symptom Assessment Score (ESAS) was used for this purpose [19]. Patients reported average symptoms on the 11-point numeric rating scale (NRS 0–10), where 0 is absence of the symptom and 10 is worst possible intensity of the symptom. A change of 1 (NRS 0–10) was considered a clinically significant difference for longitudinal changes in ESAS in symptoms during the hospital stay [20]. 

### 2.3. Statistical Analysis/Considerations

Descriptive statistics were applied. Means and medians were reported for continuous variables to describe central tendencies, and standard error (SE) or range as a measure of variability. Frequencies and proportions were reported for categorical variables. The patients were classified into the groups ‘appetite loss’ or ‘no appetite loss’, based on self-reported score at admission. Patients with a self-reported score ≥ 4 (NRS 0–10) were considered to have clinically significant appetite loss. Patients with appetite loss at admission were then classified into two groups based on whether appetite improved or not during hospitalization. Improvement was defined as an increase in the appetite score by SI ≥ 1 during the hospital stay. Additionally, we investigated the frequency and increase in dose or initiation of new pharmacological treatments to understand their potential impact on appetite improvement. 

To compare the groups and examine associations, an independent samples *t*-test was applied for continuous variables. Levene’s test was used to assess the homogeneity of variance between the groups. The chi-square test was used to compare categorical variables, while Fisher’s exact test was utilized for small sample sizes, defined as fewer than 10 patients. Symptoms included in the analysis were depression, nausea, average and worst pain, obstipation, fatigue, dyspnea and anxiety, based on their perceived impact on appetite [5]. The level of significance was a two-sided *p*-value ≤ 0.05. Missing data were mainly handled by pairwise deletion, except for missing values at discharge, where a last-value-carried-forward approach was used if a corresponding value had been recorded prior to discharge. SPSS version 29.0.1.0 (SPSS, Armonk, NY, USA) was used for data analysis.

### 2.4. Ethics

Both the primary study and the secondary analysis were classified as healthcare improvement by the Regional Committee for Medical Research Ethics, Health Region Central Norway (REK) (2018/925/REK midt and 2021/212312/REK midt), and thus, according to Norwegian health care legislation, explicit informed consent from the patients was not needed.

## 3. Results

### 3.1. Inclusion

**Figure 1:**
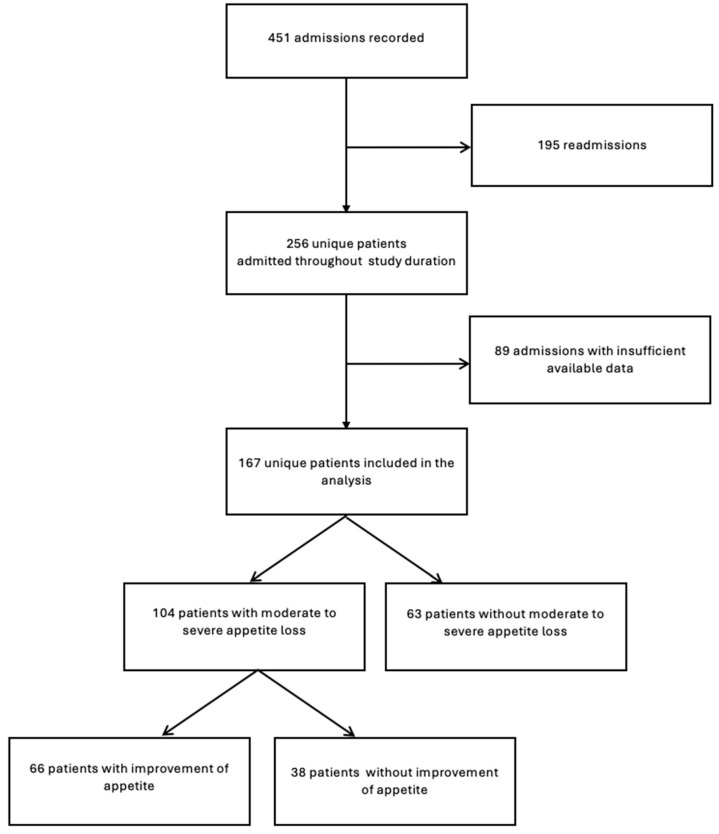
Inclusion and exclusion of patients.

Figure 1 shows the inclusion of patients. In the primary study [17], a total of 451 admissions were documented throughout the year-long study period. Of those, 195 were readmissions and subsequently excluded from the analysis. Furthermore, out of the 256 unique patients, 85 patients were excluded due to incomplete PROMs (*n* = 34), reduced general condition (*n* = 22), reduced cognitive function (*n* = 18), declined registration (*n* = 6), forms not handed out (*n* = 2), unknown reasons (*n* = 2) and not able to read Norwegian (*n* = 1) [17]. In addition, four patients were excluded due to errors in patient registration. This left 167 unique patients for the analysis.

### 3.2. Patient Characteristics 

Baseline demographics, symptoms and disease characteristics of patients with and without appetite loss are described in Table 1. At admission, 104 (62%) patients reported appetite loss. Patients living alone or with gastrointestinal (GI) cancer were more prone to experience appetite loss. Conversely, patients with breast cancer were more represented in the group without appetite loss. More patients included in the palliative care pathway experienced appetite loss compared to those in the integrated care pathway. Patients with appetite loss also had slightly poorer mean ECOG performance status score. Nausea, fatigue, depression, anxiety and dyspnea were significantly increased in the group with appetite loss. Mean hospital length of stay was 7.5 days (range 1–35). 

### 3.3. Change in Appetite during Hospitalization

Of the 104 patients who experienced appetite loss at admission at the APCU, 66 (63%) had an improvement during their hospital stay. Table 2 illustrates the disparity and similarities between these two groups. Of those who had improvement, the mean decrease in NRS score was 3.4 (SE 0.27). The group without improvement had a mean increase in NRS score of 0.87 (SE 0.22). Living alone before admission was the only factor significantly associated with improving appetite during hospital stay (*p* = 0.035).

### 3.4. Additional Symptoms

Table 3 presents the change in appetite-related symptoms for the 104 patients with appetite loss at admission, based on whether they experienced appetite improvement during their hospital stay. Patients who improved their appetite showed significant improvement in fatigue, while there was no significant parallel improvement in other symptoms.

### 3.5. Pharmacological Interventions

Table 4 details the pharmacological interventions administered during hospitalization to patients who experienced improved appetite and those who did not. We specifically investigated dose increases or the initiation of new treatments. There was no statistically significant difference in reported modifications in pharmacological interventions between the two groups, except for rehydration (*p* = 0.022), which was negatively associated with improvement in appetite.

## 4. Discussion

Nearly two thirds of the patients admitted to the APCU experienced moderate to severe appetite loss. These patients had a higher burden of additional symptoms, poorer performance status and were more frequently allocated to the palliative care pathway. Additionally, patients with GI cancer had a higher probability of appetite loss. Patients living alone had more appetite loss at admission, but also an increased likelihood for improvement. Although the majority of patients with appetite loss at admission improved their appetite during their hospital stay, living alone and not receiving rehydration during hospitalization were the only two factors potentially associated with such an improvement.

The high proportion of study patients experiencing appetite loss aligns with previous studies reporting that this is a common complaint among patients with advanced cancer [6,21]. Still, a high proportion of patients in this study showed improvement in their appetite. Early recognition of patients with appetite loss may encourage implementation of interventions to limit further progression of the symptom. With the anticipated increase in cancer prevalence and the trend of patients living longer with advanced disease [22], gaining more knowledge on managing appetite loss will become increasingly important due to the substantial benefits it offers.

GI cancer was identified as a risk factor for appetite loss, which is in line with previous studies [23,24]. GI cancer is among the most common cancer diseases worldwide [24,25], and nearly half of the patients in our study had GI cancer. This underscores the importance of focusing on appetite loss in this patient group. In contrast, our sample suggests that patients with advanced breast cancer usually have preserved appetite. Previous research has reported a weak negative association between breast cancer and weight loss [7], which may be related to preserved appetite. However, many patients with breast cancer, particularly in other phases in their disease trajectory, undergo chemotherapy, a treatment known to frequently cause appetite loss. Therefore, appetite loss should not be entirely overlooked in these patients [26].

In line with previous research, patients with appetite loss at admission also had a high burden from additional symptoms. Loss of appetite may be accompanied by or precede the development of cachexia [27]. Consequently, early recognition and implementation of measures will be of great interest for patients to avoid progression towards cachexia [28]. Patients with appetite loss also had slightly poorer performance status and were more common in the palliative care pathway, as seen in other studies, all suggesting more advanced cancer [29,30]. Although appetite loss was more frequent in the palliative care pathway, patients in the integrated care pathway undergoing anticancer treatment also experienced appetite loss. This may be due to anticancer treatment side effects such as nausea and malaise [31]. Furthermore, our study disclosed disparities in rehydration practice. In terminally ill patients, rehydration is controversial, but may prevent renal failure, accumulation of medications and restlessness [32]. The negative associations between rehydration and appetite improvement in our sample might be partly attributed to the characteristics of the study population. Additionally, previous research has shown a close relationship between thirst and hunger, which might explain why patients that did not improve their appetite more frequently also received rehydration. This may be attributed to a reduced fluid intake peri-prandially, which is when fluid consumption usually is at its highest [33,34].

In the main study published from this cohort, significant improvements in all symptoms during the hospital stay were reported [17]. This current analysis investigated the association between appetite and other symptoms. In relation to appetite improvement, fatigue was the only symptom with significant concurrent improvement. Fatigue and appetite have previously been shown to be closely related potentially due to a simultaneous response to proinflammatory cytokines [35,36]. Systemic inflammation can alter central nervous system (CNS) function, affecting the hypothalamus and consequently influencing both appetite regulation and fatigue [37]. In patients with advanced cancer, symptoms regularly appear in clusters [38], and fatigue and appetite are commonly paired [39].

In our sample, patients who were living alone showed a higher likelihood of experiencing appetite loss at admission compared to those who were living with a spouse or cohabitant. This observation aligns with another study indicating that patients often seek support in matters of nutrition within their own close circle, where a cohabitant usually plays an important part [40]. Interestingly, living alone emerged as the sole significant characteristic associated with patients experiencing improved appetite during hospitalization. These findings suggest that the provision of prepared and served meals during hospitalization may positively influence eating habits compared to preparing meals independently. However, it also indicates that eating and drinking encompass various essential dimensions that extend beyond mere nutrition [40]. Reduced food intake and weight loss may lead to a sense of helplessness or failure, loss of independence, social isolation and conflicts with family members regarding food [41]. In the modern society, shared meals serve as a means to establish and nurture relationships [42]. Therefore, the social aspect of eating is crucial when addressing appetite loss. In addition to the social aspect, weight loss will physically alter appearance, which can have a profound impact on body image and identity. Psychosocial interventions could be beneficial for the patient’s quality of life and the well-being of close family members [41]. Our findings imply that assistance in preparing food and initiatives like shared meals with other patients, caregivers and/or healthcare professionals may be beneficial during hospitalization. In addition, information about appetite loss and cachexia might also be effective to reduce the perceived stigma of the situation. An intriguing question arises regarding whether appetite improvement can be sustained after discharge when patients once again must eat alone.

As of today, several interventions for appetite loss are available, both pharmacological and non-pharmacological. Non-pharmacological interventions include dietary counseling, and assistance in reducing eating-related distress [31]. Small, energy-dense meals that look appetizing may help many patients and their next of kin during mealtimes. Regarding pharmacological interventions, numerous drugs are currently in use or undergoing evaluations. In the present analysis, no significant association was found between improved appetite and a dose increase or initiation of pharmacological interventions during hospital stay. Megestrol acetate, corticosteroids, anamorelin, olanzapine, and cannabinoids are listed as drugs with orexigenic potential alongside non-pharmaceutical interventions [43]. Corticosteroids are a well-established treatment in cancer care targeting a variety of symptoms like nausea, fatigue, dyspnea, weight loss and appetite loss [35,44]. Its anti-inflammatory effects stimulate appetite in the first weeks of use, but the effect is not lasting [6,44,45]. Additionally, long-term use of corticosteroids is usually associated with adverse effects like osteoporosis, sarcopenia, muscle loss, adrenal suppression, Cushing’s syndrome, psychiatric symptoms, immunosuppression and decreased efficiency [46,47]. Therefore, corticosteroids may offer value to patients with a limited life expectancy or as a temporary solution, but might not be advantageous for those with a longer life expectancy, especially if the primary aim is to enhance appetite in the long term [46]. In the current study, there were no significant trends between an increase or initiation of corticosteroid therapy and improvement of appetite. It is important to remember that the current study was not designed to evaluate the effect of the interventions provided, and information regarding the indication for the intervention or the dose administered was not available. The majority of patients starting or increasing the dose of corticosteroids improved their appetite. Still, an equal proportion of patients without change of corticosteroid treatment also experienced appetite improvement. This may indicate that appetite improved due to other reasons such as the alleviation of other symptoms, or the psychosocial benefits of admission, and that corticosteroid treatment was not necessary for appetite improvement. Consequently, this reminds us to always consider several different approaches to alleviate appetite loss.

Studies exploring the use of olanzapine to treat appetite loss in cancer over the last years have shown promising results [27,46,48]. Even a low dose of olanzapine demonstrates appetite-stimulating effects by targeting various receptors linked with appetite regulation [48]. However, olanzapine was only recently recommended in guidelines for treating appetite loss, and was therefore not frequently used as an appetite stimulant at the time of recruitment in the present study [49]. In line with the development of more clinical practice guidelines for cachexia in the last decade, the focus on treating appetite loss in cancer has increased. Patients with longer life expectancy may benefit from improving their appetite accompanied by enhanced nutritional intake, aiming to optimize the patient’s weight, physical function, quality of life and survival. However, improvement of appetite may, in itself, contribute to participation in meals and social events, beneficial to both the patients and their next of kin [6]. Consequently, patients reaching the end of life may also benefit from improving their appetite, even if weight remains unaltered.

### Appraisal of Methods

This paper presents a secondary analysis of a study aimed at evaluating interventions and symptom relief for patients with incurable cancer at an APCU [17]. As a result, the data acquisition was not specifically tailored to the current study, leading to design limitations such as missing information regarding the specific interventions against appetite loss. Furthermore, there was no information regarding other concurrent symptoms such as early satiety, taste or smell changes, food aversions or diurnal variations in appetite. Additionally, crucial details such as the indication for the medications, specific orexigenic medications, and information on patients already using medications were not included in this secondary analysis. Variations in the length of hospital stays may result in varying time frames between the first and last PROMs assessments. Consequently, the effect of initiated interventions may not always have reached its full potential during hospitalization. Furthermore, the study’s limitations also include the absence of data from patients with hematological, lung and gynecologic cancer. Moreover, the results may not be generalizable to other patient populations, as the patients admitted to an ACPU are selected patients with advanced cancer and a high symptom burden. In the analysis regarding pharmacological interventions, it is difficult to prove which therapies caused the improvement, as multiple interventions were carried out simultaneously at the APCU. Pairwise deletion was utilized to handle missing data, but may introduce biased estimates, loss of valuable information and reduced statistical power due to the exclusion of cases with missing values. To mitigate this, we used last value carried forward where available. However, this was of course not possible for all patients with missing data, and the method does not completely resolve bias as it is especially known to lead to conservative estimates [50].

## 5. Conclusions

The majority of patients admitted to the APCU had appetite loss. A significant number of these patients showed improvement in their appetite during their hospital stay. Interestingly, patients living alone were identified as being at risk of having appetite loss at admission, and they were also the single identifiable patient group with a significant improvement in appetite during hospital stay. Our findings indicate that assessing living conditions is an important part of a holistic assessment for customizing interventions to address appetite issues.

## Figures and Tables

**Table 1:** Baseline clinical characteristics and demographics at admission.

	Appetite Loss ^a^ *n* = 104	No Appetite Loss *n* = 63	*p*-Value
Appetite score at admission, mean ^b^ (SE ^c^)	6.6 (0.19)	1.2 (0.15)	
Age, mean (range)	71 (29–92)	69 (40–89)	0.179
Gender, *n* (%)			0.866
Male	68 (65)	42 (67)	
Female	36 (35)	21 (33)	
Living situation, *n* (%)			**0.024**
Living alone	45 (43)	16 (25)	
Married or cohabitant	56 (54)	45 (71)	
Cancer diagnosis, *n* (%)			
Gastrointestinal	51 (49)	20 (32)	**0.028**
Urological	26 (25)	17 (27)	0.776
Breast	3 (3)	8 (13)	**0.021** ^d^
Head and neck	5 (5)	6 (10)	0.335 ^d^
Others	19 (18)	12 (19)	0.900
ECOG ^e^ Performance status, mean (SE ^c^)	2.5 (0.08)	2.3 (0.09)	**0.013**
Care pathway, *n* (%)			**0.019**
Palliative care pathway	68 (65)	30 (48)	
Integrated care pathway	35 (34)	33 (52)	
Additional symptoms, mean ^b^ (SE ^c^)			
Nausea	2.5 (0.25)	1.4 (0.27)	**0.004**
Average pain	3.6 (0.25)	4.1 (0.36)	0.234
Worst pain	4.8 (0.29)	5.6 (0.42)	0.125
Obstipation	3.1 (0.32)	2.7 (0.39)	0.467
Depression	3.9 (0.26)	2.5 (0.32)	**0.001**
Fatigue	6.2 (0.21)	4.4 (0.33)	**0.001**
Dyspnea	3.6 (0.26)	2.4 (0.33)	**0.005**
Anxiety	3.2 (0.26)	2.4 (0.30)	**0.043**

^a^ Numeric rating scale (NRS) ≥ 4 at admission of a NRS scale 0–10, where 0 is absence of the symptom and 10 is worst possible. ^b^ Mean = mean score of a NRS scale 0–10. ^c^ SE = standard error. ^d^ *p*-value from Fisher’s exact test. ^e^ ECOG = Eastern Cooperative Oncology Group. Bold = significant values.

**Table 2:** Appetite development during hospitalization in the group with appetite loss at admission.

	Appetite Improvement ^a^, *n* = 66	No Appetite Improvement, *n* = 38	*p*-Value
Appetite score at admission, mean ^b^ (SE ^c^)	6.7 (0.24)	6.5 (0.29)	0.531
Age, mean (range)	72 (29–92)	69 (40–89)	0.102
Gender, *n* (%)			0.075
Male	39 (59)	29 (76)	
Female	27 (41)	9 (24)	
Living situation, *n* (%)			**0.035**
Living alone	34 (52)	11 (29)	
Married or cohabitant	31 (47)	25 (66)	
Cancer diagnosis, *n* (%)			
Gastrointestinal	36 (55)	15 (40)	0.139
Urological	13 (20)	13 (34)	0.100
Breast	3 (5)	0 (0)	0.298 ^d^
Head and neck	2 (3)	3 (8)	0.352 ^d^
Others	12 (18)	7 (18)	0.976
ECOG Performance status, mean (SE ^c^)	2.5 (0.09)	2.6 (0.13)	0.890
Care pathway, *n* (%)			0.183
Palliative care pathway	68 (65)	30 (48)	
Integrated care pathway	35 (34)	33 (52)	

^a^ Improvement > 1 on numeric rating scale (NRS). ^b^ Mean = mean score of a NRS scale 0–10, where 0 is absence of the symptom and 10 is worst possible. ^c^ SE = standard error. ^d^ *p*-value from Fisher’s exact test. Bold = significant values.

**Table 3:** Associations between improvement of appetite and improvement of other symptoms.

	Improvement of Appetite, *n* = 66	No Improvement of Appetite, *n* = 38	*p*-Value
Symptom Improvement ^a^, *n* (%)			
Nausea	29 (44)	12 (32)	0.214
Average pain	37 (56)	18 (47)	0.348
Worst pain	33 (50)	17 (45)	0.683
Obstipation	31 (47)	19 (50)	0.896
Depression	28 (42)	13 (34)	0.342
Fatigue	47 (71)	17 (45)	**0.005**
Dyspnea	34 (52)	17 (45)	0.438
Anxiety	31 (47)	15 (40)	0.529

^a^ Number of patients with improvement of numeric rating scale (NRS ≥ 1) on Edmonton Symptom Assessment Score (ESAS) for each individual symptom. Bold = significant values.

**Table 4:** Associations between improvement of appetite and dose increase ^a^ in pharmacological interventions.

	Improvement of Appetite, *n* = 66	No Improvement of Appetite, *n* = 38	*p*-Value
Medication, *n* (%)			
Corticosteroids	26 (39)	15 (40)	0.994
Benzodiazepines (incl. hypnotics)	15 (23)	9 (24)	0.911
Laxatives	23 (35)	17 (45)	0.318
Antiemetics	12 (18)	13 (34)	0.065
Medications for dyspnea	5 (8)	1 (3)	0.412 ^b^
Medications for insomnia	8 (12)	3 (8)	0.742 ^b^
Rehydration	28 (42)	25 (66)	**0.022**

^a^ Including patients who started on a medication and patients with an increase of dose of an already established treatment. ^b^ *p*-value from Fisher’s Exact Test. Bold = significant values.

## Data Availability

The datasets generated during and/or analyzed during the current study are available from the corresponding author upon reasonable request.
